# The Anticoagulated trauma patient in the age of the direct oral anticoagulants: a Canadian perspective

**DOI:** 10.1186/s13049-017-0420-y

**Published:** 2017-08-02

**Authors:** Brendan Wood, Barto Nascimento, Sandro Rizoli, Michelle Sholzberg, Amanda McFarlan, Andrea Phillips, Alun D. Ackery

**Affiliations:** 1 Division of Emergency Medicine, Department of Medicine, University of Toronto, Toronto, Canada; 2 Department of Surgery, Sunnybrook Health Sciences Centre, 2075 Bayview Avenue, Toronto, Canada; 3 Department of Surgery, St Michael’s Hospital, 30 Bond Street, Toronto, Canada; 4 Department of Medicine and Laboratory Medicine & Pathobiology, St Michael’s Hospital, Li Ka Shing Knowledge Institute, University of Toronto, Toronto, Canada; 5 Department of Emergency Medicine, St Michael’s Hospital, 30 Bond Street, Toronto, Canada

**Keywords:** Trauma, Direct oral anticoagulants, Warfarin, Dabigatran, Rivaroxaban, Apixaban

## Abstract

**Background:**

The anticoagulated trauma patient presents a particular challenge to the critical care physician. Our understanding of these patients is defined and extrapolated by experience with patients on warfarin pre-injury. Today, many patients who would have been on warfarin are now prescribed the Direct Oral Anticoagulants (DOACs) a class of anticoagulants with entirely different mechanisms of action, effects on routine coagulation assays and approach to reversal.

**Methods:**

Trauma registry data from Toronto’s (Ontario, Canada) two Level 1 trauma centres were used to identify patients on oral anticoagulation pre-injury from June 1, 2014 to June 1, 2015. The trauma registry and medical records were reviewed and used to extract demographic and clinical data.

**Results:**

We found 81 patients were on oral anticoagulants pre-injury representing 3.2% of the total trauma population and 33% of the orally anticoagulated patients were prescribed a DOAC prior to presentation. Comparison between the DOAC and warfarin groups showed similar age, mechanisms of injury, indications for anticoagulation, injury severity score and rate of intracranial hemorrhage. Patients on DOACs had higher initial mean hemoglobin vs warfarin (131 vs 120) and lower serum creatinine (94.8 vs 129.5). The percentage of patients receiving a blood transfusion in the trauma bay and total in-hospital transfusion was similar between the two groups however patients on DOACs were more likely to receive tranexamic acid vs patients on warfarin (32.1% vs 9.1%) and less likely to receive prothrombin concentrates (18.5% vs 60%). Patients on DOACs were found to have higher survival to discharge (92%) vs patients on warfarin (72%).

**Conclusion:**

Patients on DOACs pre-injury now represent a significant proportion of the anticoagulated trauma population. Although they share demographic and clinical similarities with patients on warfarin, patients on DOACs may have improved outcomes despite lack of established drug reversal protocols and challenging interpretation of coagulation assays.

Level of Evidence: III; Study Type: Retrospective Review.

## Background

Trauma patients who are on oral anticoagulation pre-injury are a vulnerable subset of the trauma population and account for approximately 4% of patients presenting with traumatic injury [1]. These patients tend to be older and have multiple medical comorbidities. Furthermore, pre-injury anticoagulation has been shown to be an independent predictor of mortality [2, 3].

Current literature and management strategies are based on experience with patients on Vitamin K antagonists (e.g. warfarin). In the last several years the direct oral anticoagulants (DOACs) including dabigratan etexilate (dabigatran) a direct thrombin inhibitor as well as rivaroxaban, apixaban and edoxaban which are direct factor Xa inhibitors have reached the market. Large randomized control trials studying these agents for stroke prophylaxis as well as prophylaxis and treatment of venous thromboembolic disease (VTEs) have established improved safety and similar efficacy compared to warfarin [4–10]. These results combined with standardized dosing and elimination of blood monitoring have led to the widespread use of DOACs in clinical practice.

Although promising, use of DOACs pose challenges to acute care providers from both an assessment and management perspective. Due to their mechanism of action, DOAC use is associated with non-dose dependent effects on standard coagulation assays, such as the activated partial thromboplastin time (aPTT) and prothrombin time (PT) or international normalized ratio (INR) [11]. When initially approved, a major concern was the lack of a specific reversal agent for instances of life-threatening hemorrhage or emergency surgery. Idarucizumab, a dabigatran reversal agent has recently been approved for use and direct Xa inhibitor reversal agents are expected to be approved in the near future. However, their clinical effectiveness and rational use in the setting of trauma is not yet well characterized.

As increasing numbers of the general population are put on DOACs, an increasing number of trauma patients who would have previously been on warfarin pre-injury will be presenting to trauma services on DOACs. Here we present data from a retrospective cohort using two trauma registries to characterize the clinical characteristics of the anticoagulated trauma patient population presenting to our Toronto-based trauma centres.

## Methods

Participants were identified using the trauma registries at St. Michael’s Hospital (SMH) and Sunnybrook Health Sciences Centre (SHSC) which are both Urban, Level 1 trauma centres in Toronto, Canada. The trauma registries at both sites collect standardized data in-line with the American College of Surgeons Trauma Quality Improvement Program (TQIP) guidelines which are widely used in over 450 sites across North America. Demographic data and clinical outcomes for all patients admitted to the trauma service at SMH and SHSC are systematically tracked and maintained in validated databases. Both registries are routinely reviewed by the Canadian Institute of Health Information (CIHI) and the National Trauma Databank in the United States. Both systems have data validators that are used to ensure accuracy of the Registry Database; in addition, internal monthly, quarterly and annual data quality and inter-rater reliability reviews are performed to ensure data accuracy and reliability. Patients were identified by searching for trauma patients presenting from June 1, 2014 to June 1, 2015 who were identified as having a “bleeding disorder” which included oral anticoagulation or if they had atrial fibrillation as a comorbidity. These charts were screened by a trained reviewer to verify pre-injury oral anticoagulant prescription and then demographic and clinical data was extracted from the trauma registry dataset and the electronic medical record. Descriptive statistics were performed with Microsoft Excel. This study was reviewed and approved by the Sunnybrook Health Sciences Centre Research Ethics Board (076–2015) and the St. Michael’s Hospital Research Ethics Board (15–027). No patient consent was needed to collect information from both institutions' trauma registries.

## Results

From June 1, 2014 to July 31, 2015, we identified 81 patients taking pre-injury oral anticoagulants which represented 3.2% of the total trauma registry entries for this time period. Of these patients, 67% were prescribed warfarin and 33% were prescribed DOACs (Dabigatran 12%; Rivaroxaban 12%; Apixaban 9%). The mean age of patients taking DOACs versus warfarin respectively was 77.5 vs. 75.6; 37.0% vs. 55.6% were male and 3.7% vs. 5.5% had concomitant prescription of antiplatelet agents (Table 1). For both groups the most common indication for oral anticoagulation was atrial fibrillation (DOACs 74% vs. warfarin 74%) and the most common mechanism of injury was fall (DOACs 55.5% vs warfarin 76%). Patients on DOACs had similar rates of intracranial hemorrhage (ICH) compared to warfarin (70.0% vs. 66.7% *p* = 0.74), Injury Severity Score (ISS) (16.6 vs 20.5 *p* = 0.06) and Glasgow Come Scale (GCS) (12.9 vs 12.2 *p* = 0.49). There was a difference in survival to discharge (DOACs: 92%, warfarin: 72% *p* = 0.03) (Table 2). Patients on DOACs were less likely to receive prothrombin complex concentrates (PCC) (DOACs: 18.5% vs Warfarin 60% *p* = 0.05), received tranexamic acid more frequently (32.1% vs 9.1% *p* = .01) and had similar rates of packed red blood cell transfusion (pRBC) in the trauma bay (DOACs: 10.7% vs Warfarin 7.3% *p* = 0.6) and total average pRBC transfusion while hospitalized (1.1 units vs 0.82 units *p* = 0.72). Patients on DOACs had higher initial hemoglobin values (DOACs: 131 vs. warfarin:120 *p* = .03) and lower serum creatinine (94.8 vs 129.5 *p* = 0.17). 66.7% of patients on warfarin were found to have an international normalized ratio’s (INR) greater than 1.5 whereas 33.3% of patients on Anti-Xa inhibitors had INRs greater than 1.5 and 54.5% of patients on Dabigatran had activated partial thromboplastin times (aPTT) greater than 35 s (Table 3).

**Table 1:** Demographic & clinical characteristics of orally anticoagulated trauma patients

	Warfarin	DOACs
Age	77.5	75.6
Male Gender	55.6%	37%
Indication for anticoagulation		
Atrial fibrillation	76%	74%
Thromboembolism	3.7%	0
Prosthetic heart valves	1.9%	0
Unknown	18.5%	16%
Clopidogrel use	5.5%	3.7%
Mechanism of Injury		
Motor vehicle collision	14.8%	18.5%
Fall	76%	55.5%
Pedestrian Struck	3.7%	14.8%
Penetrating	0	3.7%
Other	5.6%	7.4%

**Table 2:** Injury scores, outcomes & lab values for orally anticoagulated trauma patients

	Warfarin	DOACs	*P* value
ISS	20.5	16.6	0.06
GCS	12.2	12.9	0.49
ICH	66.7%	70%	0.74
Survival to Discharge	72%	92%	0.03
Hemoglobin	120	131	0.03
Serum Creatinine	129.5	94.8	0.17

*ISS* Injury severity score, *GCS* Glasgow coma scale, *ICH* Intracranial hemorrhage

**Table 3:** Blood product & coagulation assays for orally anticoagulated trauma patients

	Warfarin	DOACs	Dabigatran	Anti Xa inhibitors	*p*-value
Trauma bay pRBC transfusion %	7.3%	10.7%	-	-	0.6
Total pRBC transfusion	0.82 units	1.1 units	-	-	0.72
TXA administration	9.1%	32.1%	-	-	0.01
PCC administration	60%	18.5%	-	-	0.05
INR >1.5	66.6%	-	-	33.3%	-
aPTT >35 s	-	-	54.5%	-	-

*pRBC* packed red blood cells. *TXA* tranexamic acid. *PCC* prothrombin complex concentrates. *INR* international normalized ratio. *PTT* activated partial thromboplastin time

## Discussion

Although DOACs have been widely adopted in clinical practice there is a paucity of studies examining the effects of DOAC use in the Canadian trauma population. In addition, to our knowledge, this study is the first to examine transfusion and coagulation assay data in orally anticoagulated trauma patients since adoption of the DOACs. We have characterized a shift in oral anticoagulant therapy in trauma patients; a third of patients studied were on DOAC therapy. We suspect that this trend will continue as use of DOACs continues to rise.

In our study the warfarin group and the DOAC group had similar rates of pRBC transfusion on both initial presentation and cumulatively while in hospital however there were differences in use of hemostatic agents namely PCC and TXA. Since approval, caution against the use of DOACs has related to the lack of a specific reversal agent to manage life-threatening hemorrhage or need for emergent surgeries [12]. In lieu of a specific reversal agent, recommendations have been centered on supportive care, general hemostatic measures and judicious use of factor concentrates which have been shown to reduce anticoagulant activity in healthy individuals and ex-vivo studies [13]. The clinical benefit of such interventions however, is not well studied and in our practice setting there is considerable ambivalence regarding their use. The RE-VERSE AD trial studied the effects of giving Idarucizumab, a monoclonal antibody against free and thrombin bound dabigatran, to patients with life-threatening hemorrhage including “trauma-related” or requiring urgent surgery taking dabigatran and found that idarucizumab caused rapid reversal of anticoagulation [14]. The ANNEXA-A and ANNEXA-R trials studied the effects of giving andexanet alpha, an anti-factor Xa decoy protein, to healthy volunteers taking apixaban or rivaroxaban and was found to rapidly reverse anticoagulation [15]. Idarucizumab was recently approved for clinical use by the US Food and Drug Administration and Health Canada. Andexanet alpha is currently being studied in patients with bleeding on Factor Xa Inhibitors. It remains to be seen how introduction of these agents will affect clinical practice and patient outcomes such as mortality and need for transfusion.

Our findings related to lower mortality with DOACs are similar to findings by Maung et al., who recently published a large retrospective study using trauma registry data also comparing patient on DOACs vs. warfarin [16]. Other literature comparing mortality for bleeding patients taking DOACs have come from sub-group analysis of the RE-LY trial as well as series of patients suffering all-cause ICH. Sub-group analysis of the RE-LY trial found that mortality for combined spontaneous and traumatic ICH in patients was similar for patients taking dabigatran versus warfarin although the overall event rate was lower in the dabigatran group [17]. Alonso et al. [18] reported a large series of all-cause ICH in patients taking either dabigatran or warfarin derived from healthcare databases and found no difference in mortality between the two groups. Our study and Maung et al. included both intra- and extracranial injuries and excluded spontaneous ICH. It is possible that selection bias due to the inclusion of patients with extracranial injury affected outcomes. Another factor which may have affected mortality is anticoagulant activity at time of injury, in our study a greater proportion of patients taking VKAs had abnormal coagulation assays versus the DOAC group although these assays have limited utility when assessing anticoagulant activity induced by DOACs.

Our study does have limitations that must be considered when evaluating our results. The trauma registries used are not specifically designed to survey oral anticoagulant use and it is possible that study participants have been missed, however our finding of 3.2% oral anticoagulant exposure in trauma patients is in-line with previous publications [1]. Our dataset was not able to capture time since last medication ingestion and given the shorter half-life of the DOACs, some patients may have had sub-therapeutic serum drug levels at time of assessment and thus lower risk of bleeding. Advanced coagulation studies such as the dilute-thrombin time (Hemoclot assay) and drug specific anti-Xa assays are available at some of our institutions but are not part of routine “trauma blood work” and are available only in consultation with the hematology service. Regular use of these assays would have allowed for definitive evaluation of anticoagulant activity at time of injury. We have however, reported the proportion of patients with abnormal “routine” coagulation test results as a surrogate of anticoagulant activity, although we recognize their limited reliability in the detection of DOAC drug presence (i.e. a normal test result does not definitively rule out DOAC presence).

## Conclusion

Our study found that trauma patients taking DOACs pre-injury shared many clinical similarities with patients taking VKAs including indication for anticoagulation, mechanism of injury, presence of ICH, ISS and GCS. We did find that mortality was lower in patients taking DOACs pre-injury, however the percentage of patients with abnormal coagulation assays was lower in the DOAC group. We believe ongoing surveillance by trauma registries and ongoing studies are needed in this area as our ageing population are increasingly prescribed DOACs. Future studies which incorporate advanced coagulation assays to definitively measure anticoagulant activity and assessment of benefit of new reversal agents would be particularly valuable.
